# Lipoprotein Lipase/Apolipoprotein Cll Gene Polymorphism in Kurdish Patients With Severe Hypertriglyceridemia

**DOI:** 10.7759/cureus.46829

**Published:** 2023-10-11

**Authors:** Kajeen Hussein, Sherwan Salih, Dhia Al-Timimi

**Affiliations:** 1 Department of Medical Chemistry, College of Medicine, University of Duhok, Duhok, IRQ

**Keywords:** kurdish population, apolipoprotein cll, lipoprotein lipase, hypertriglyceridemia, genotyping

## Abstract

Background

Polymorphisms in the *lipoprotein lipase* (*LPL*) and *apolipoprotein CII (APO CII)* genes have been linked to severe hypertriglyceridemia in several populations. This study investigated the frequency of *LPL-*Hind lll* *and* APO Cll-*Ava ll polymorphism among Kurdish patients with severe hypertriglyceridemia.

Methodology

We investigated *LPL-*Hind llland *APO Cll*-Ava ll gene polymorphism in a sample of Kurdish patients receiving treatment at Azadi Teaching Hospital in Duhok, Kurdistan Region, Iraq. We included a total of 100 subjects in this study, of which 64 had severe hypertriglyceridemia, and 36 had normotriglyceridemia. There were 56 males and 44 females. We used the polymerase chain reaction-restriction fragment length polymorphism technique to determine the polymorphism of the *LPL-*Hind lll and *APO Cll-*Ava ll genes.

Results

In those with severe hypertriglyceridemia, the most frequent alleles were H+H+ *LPL-*Hind lll polymorphism (42, 65.6%) followed by A1A1 *APO Cll-*Ava ll polymorphism (30, 46.9%), whereas these frequencies were 16 (44.4%) and 6 (16.7%) in those with normotriglyceridemia, respectively. The H+H+ genotype group had considerably higher triglyceride levels and lower high-density lipoprotein cholesterol levels compared with the H−H− genotype group. A similar pattern was observed when comparing the A1A1 and A2A2 genotype groups, with both patterns being statistically significant.

Conclusions

Our results showed a high frequency of H+H+ *LPL-*Hind III polymorphism in those with hypertriglyceridemia, which may be a hereditary indicator of vulnerability to this condition in the Kurdish population.

## Introduction

Hypertriglyceridemia is a common biochemical disorder that can result from primary or, more commonly, secondary causes [1]. Hypertriglyceridemia is an independent risk factor for cardiovascular disease (CVD) [2]. High levels of serum total cholesterol (TC), triglycerides (TGs), very-low-density lipoprotein cholesterol (VLDL-c), low-density lipoprotein cholesterol (LDL-c), and high-density lipoprotein cholesterol (HDL-c) are all hallmarks of atherogenic dyslipidemia [3]. Lipoprotein lipase (LPL) is a multifunctional enzyme that is crucial to lipid metabolism, as it converts TG into lipoprotein molecules in the circulation. This conversion is regarded as a limiting step in the removal of lipoproteins, endogenous VLDL-c, and exogenous chylomicrons [4].

Both the LPL enzyme and its essential activator, apolipoprotein CII (APO CII), act on substrate lipoproteins [5]. Residing on chromosome 8p22, the* LPL* gene has nine introns and 10 exons and is approximately 35 kb in size. The human *APO CII *gene is found on chromosome 19 at the long arm (19q13.2) of the human *APO E-APO A-APO CII* gene cluster [6,7]. Nearly 100 mutations and single-nucleotide polymorphisms have been discovered in the human *LPL* gene through genetic studies [8,9]. Small changes in serum lipid levels have been observed with the removal of the Hind lll restriction enzyme recognition site, caused by the replacement of the thymine (T) base with the glutamine (G) base at position +495 of a Hind lll polymorphism on intron 8 [10,11]. However, little is known about the *LPL*/*APO CII* gene polymorphism in the Kurdish population, especially among those with severe hypertriglyceridemia. As severe hypertriglyceridemia is an established risk factor of CVD, it is important to investigate the distribution of *LPL*/*APO CII* gene polymorphism in the Kurdish population, as this polymorphism might provide insight into the nature of the disease. Therefore, in this study, we investigate the frequency of *LPL*-Hind III and *APO CII*-Ava II polymorphism in patients with severe hypertriglyceridemia not suffering from any secondary cause, as well as a group of normotriglyceridemia subjects.

## Materials and methods

Study population

We obtained a retrospective sample using a random sampling procedure for patients with severe hypertriglyceridemia who were referred to the Endocrinology Department at Azadi Teaching Hospital in Duhok, Kurdistan Region, Iraq, between January 2021 and March 2022. We registered a total of 100 volunteers for the study over six consecutive months, ranging in age from 19 to 63 years. Of these, 64 patients had severe hypertriglyceridemia with prominent signs and symptoms of CVD (TG > 500 mg/dL). The inclusion criteria were patients with hypertriglyceridemia who were not suffering from any secondary cause, did not drink alcohol, did not smoke, and had a positive family history of CVD. A control group comprised thirty-six patients with normotriglyceridemia, fasting serum TG levels below 150 mg/dL, and a negative family history of CVD. After we interviewed the participants, we explained the purpose of the study to each patient and received verbal consent.

Following an overnight fast, 5 mL of blood was collected from each subject through vein puncture. The blood was then divided into three parts: the first part (2 mL) was collected in a test tube containing ethylenediaminetetraacetic acid (EDTA) and held at −70 °C until it was used for *LPL*-Hind III and *APO Cll*-Ava ll gene polymorphism analyses; the second part (1 mL) was placed in a test tube containing EDTA for hemoglobin A1c (HbA1c) testing; and the third part (2 mL) was placed in an empty gel test tube for serum. The Cobas 6000 (open, automated, discrete, and random access; Roche, Mannheim, Germany) clinical chemistry analyzer was used to determine serum TG, HDL-c, glucose, creatinine, blood urea, and thyroid-stimulating hormone (TSH) concentrations.

Gene polymorphism analyses

**Figure 1:**
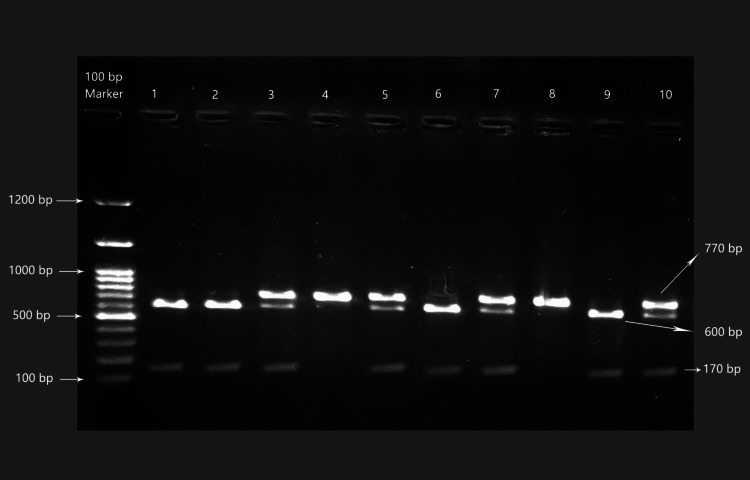
Electrophoresis of agarose gel (2.5%) with Hind lll 1200-bp DNA marker lane indicating molecular weight. Lanes 1, 2, 6, and 9 correspond to H+H+ polymorphism. Lanes 3, 5, 7, and 10 correspond to H+H− polymorphism. Lanes 4 and 8 correspond to H−H− polymorphism.

Genomic DNA purification was done at Central Lab, Azadi Teaching Hospital. A purification kit was used for genomic DNA isolation from frozen whole blood cells and placed in a tube with EDTA according to the manufacturer’s recommended protocols (AddPrep Genomic DNA Extraction Kit, Korea). The quantity and quality of the genomic DNA samples were measured using a nanophotometer (Implen NanoPhotometer [NP] 80, Implen GmbH, München, Germany) before storing them at −20 °C for further use. LPL-Hind III polymorphism was investigated by using the forward primer 5'-TGA AGC TCA AAT GGA AGA GT-3' and the reverse primer 5'-TAC AAG CAA ATG ACT AAA-3'. Polymerase chain reaction (PCR) was performed under the following conditions: 94 °C for 2 minutes of initial denaturation; then 40 cycles of 94 °C for 15 seconds, 50 °C for 30 seconds, and 72 °C for 1 minute; followed by 2 minutes of a final extension at 72 °C. Hind III (England BioLabs -Ro:105) was used to digest the PCR products. Electrophoresis was used to separate the fragments produced by the restriction enzyme. After digestion, the Hind III site generated 600-bp and 170-bp fragments, with the polymorphic allele showing the restriction site referred to as the H+ allele, and that without the site referred to as the H− allele [4]. The existence of a 600-bp band and a 170-bp band indicated the homozygous H+H+ genotype; 770-bp, 600-bp, and 170-bp bands indicated the heterozygous H+H− genotype; and a 770-bp band indicated the homozygous H−H− genotype (Figure 1).

**Figure 2:**
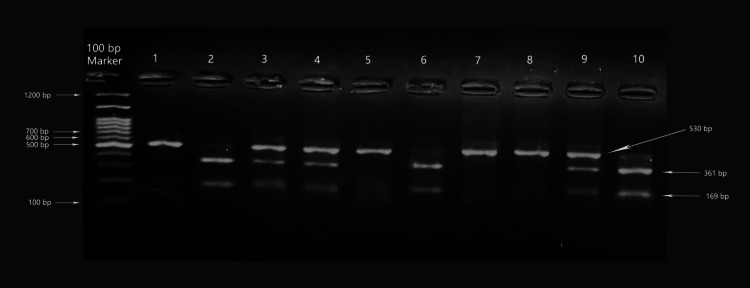
Electrophoresis of agarose gel (2.5%) for Ava ll. Lane 1 is a 200-bp DNA lane marker indicating molecular weight. Lanes 1, 5, 7, and 8 are from patients with A1A1 polymorphism. Lanes 2, 6, and 10 are from patients with A2A2 polymorphism. Lanes 3, 4, and 9 are from a patient with A1A2 polymorphism.

*APO CII*-Ava II polymorphisms were identified using the forward primer 5'-CTG CAT CCA GGA CCC AGA AGT TC-3' and the reverse primer 5'-CCT TGA GTC CTC AGA AAA GCA G-3', wherein we investigated nucleotide alteration from T to C at position 3548 of intron 3. The following conditions were used for the PCR reaction: initial denaturation at 94 °C for 2 minutes; 35 cycles of 94 °C for 30 seconds, 60 °C for 30 seconds, and 72 °C for 1 minute; and a final extension at 72 °C for 3 minutes. The PCR products were digested by the Ava II (Bio Labs Inc., New England BioLabs, Ipswich, MA) restriction enzyme. Fragment bands 361-bp and 169-bp were produced in the naturally occurring Ava II restriction site. Allele A2 refers to the polymorphic allele with the restriction site, whereas A1 refers to that without the site [4]. The existence of 361-bp and 169-bp bands indicated the homozygous A2A2 genotype; 530-bp, 361-bp, and 169-bp bands indicated the heterozygous A1A2 genotype; and a 530-bp band indicated the homozygous A1A1 genotype (Figure 2). 

Ethical approval

We obtained ethical approval for the study from the General Directorate of Health in Duhok Governorate, with document number 13072021-7-16 (including the number and the date, July 13, 2021). We also obtained informed consent from the respondents after explaining the objective of the study.

Statistical analysis

We used IBM SPSS Statistics for Windows, Version 25.0 (IBM Corp., Armonk, NY) to analyze the data. The laboratory parameter values are displayed as a mean and standard deviation (SD). We used the chi-square test to compare variables, and the student’s independent t-test to compare variables between groups. A *P*-value of ≤0.05 was considered statistically significant. To ascertain the relationship between genotypes, odds ratios (ORs) and related 95% confidence intervals (CIs) were used.

## Results

Table 1 shows the general characteristics of the participants. We found significant differences in serum TG level (*P *< 0.001) and HDL-c level (*P *= 0.001) between the normotriglyceridemia and hypertriglyceridemia groups. We found no significant differences with respect to age, body mass index, glucose, or TSH.

**Table 1:** General characteristics of study participants. The data are represented as mean ± SD. ^*^P ≤ 0.05. *N*, number; SD, standard deviation; BMI, body mass index; WC, waist circumference; SBP, systolic blood pressure; DBP, diastolic blood pressure; CRP, c-reactive protein; HDL-c, high-density lipoprotein cholesterol; HbA1c, hemoglobin A1c; TSH, thyroid-stimulating hormone

Characteristics	Subjects with normotriglyceridemia (*N *= 36) (Mean ± SD)	Patients with hypertriglyceridemia (*N *= 64) (Mean ± SD)	*P*-value
Age (years)	43.6 ± 10.5	40.2 ± 11.7	0.151
BMI (kg/m^2^)	26.7 ± 3.0	27.8 ± 2.9	0.075
WC (cm)	103.9 ± 8.3	105.1 ± 9.8	0.536
SBP (mmHg)	11.8 ± 0.7	12.4 ± 1.2	0.007^*^
DBP (mmHg)	7.7 ± 0.7	8.3 ± 1.0	0.001^*^
CRP (mg/L)	1.7 ± 1.3	3.4 ± 3.0	0.001^*^
Triglyceride (mg/dL)	105.9 ± 22.3	1001.6 ± 473.2	<0.001^*^
HDL-c (mg/dL)	44.0 ± 8.0	26.5 ± 4.3	<0.001^*^
Glucose (mg/dL)	93.8 ± 7.9	97.0 ± 11.4	0.138
HbA1c (%)	5.1 ± 0.2	5.2 ± 0.3	0.077
TSH (IU/mL)	2.1 ± 1.2	2.6 ± 1.1	0.037^*^

Table 2 shows the genotypic and allelic frequencies of the *LPL*-Hind III and *APO CII*-Ava II polymorphisms. In the case of polymorphism of the *LPL* gene, the H+H+ genotype denotes homozygotes with two alleles in which the restriction site is present; the H+H− genotype denotes heterozygotes with one allele with the site present and one allele with the site absent; and the H−H− genotype denotes homozygotes with two alleles with the restriction site absent. The H+H+ genotype was observed to be the most common allele set found in the patients with severe hypertriglyceridemia, as compared to normotriglyceridemia group.

**Table 2:** Frequency distribution of LPL-Hind lll and APO Cll-Ava II polymorphisms among study groups. Data are represented as *N *(%). ^*^P ≤ 0.05. *N*, number; LPL, lipoprotein lipase; APO Cll, apolipoprotein Cll

Study group	Genotype LPL-Hind III, *N* (%)			Genotype APO C II-Ava II, *N* (%)			Allele frequency LPL-Hind lll/APO Cll-Ava ll (%)			
	H+H+	H+H-	H-H-	A1A1	A1A2	A2A2	H+	H-	A1	A2
Patients with established hypertriglyceridemia (*N *= 64)	42 (65.6)	20 (31.2)	2 (3.2)	30 (46.9)	20 (31.2)	14 (21.9)	0.81	0.19	0.63	0.37
Patients with normotriglyceridemia (*N *= 36)	16 (44.4)	12 (33.3)	8 (22.3)	6 (16.7)	12 (33.3)	18 (50.0)	0.61	0.39	0.33	0.67
OR	2.3	80.90	0.11	4.41	0.90	0.28	0.022	0.415	0.004	0.002
*P*-value	0.022^*^	0.415	0.004^*^	0.001^*^	0.415	0.002^*^	-	-	-	-

According to the Hind III polymorphism distribution analysis, patients with severe hypertriglyceridemia had higher frequencies of the H+H+ and H+H− genotypes than those with normotriglyceridemia (96.8% vs. 77.7%, respectively). A similar pattern was observed for the A1A1 polymorphism, where the A1A1 genotype denoted homozygosity for the absence of the Ava II restriction site, whereas the A2A2 genotype denoted homozygosity for the existence of a naturally occurring Ava II restriction site. The A1A2 heterozygote genotype will exist if both alleles are present. The distribution of *APO CII* showed that the hypertriglyceridemia group had a higher frequency of the A1A1 genotype and a lower frequency of the A2A2 genotype compared to the normotriglyceridemia group, and this difference was statistically significant.

To determine which of the alleles was significantly associated with serum TG and HDL-c levels, we used the student’s t-test to compare the means between groups, as shown in Table 3. We found a significant difference in HDL-c between those with the H+H+ and H+H− alleles (*P *= 0.05). Regarding *APO Cll*-Ava ll, the A1A1 group had significantly higher TG levels compared to the A2A2 group (*P *= 0.02).

**Table 3:** Triglycerides and HDL-c levels stratified by LPL-Hind lll and APO Cll-Ava ll polymorphisms in patients with severe hypertriglyceridemia. Data are represented as mean ± SD. ^*^P ≤ 0.05. SD, standard deviation; HDL, high-density lipoprotein; LPL, lipoprotein lipase; APO Cll, apolipoprotein Cll

Genotype	Triglycerides (mean ± SD) (mg/dL)	HDL-cholesterol (mean ± SD) (mg/dL)
Hind lll		
H+H+	1016.7 ± 362.4	26.7 ± 3.8
H+H−	1022.5 ± 755.8	24.5 ± 4.9
*P*-value	0.967	0.057^*^
H+H+	1016.7 ± 362.4	26.7 ± 3.8
H−H−	841.0 ± 340.3	30.6 ± 4.7
*P*-value	0.506	0.166
Ava-ll		
A1AI	1122.0 ± 430.8	25.3 ± 4.3
AIA2	1007.8 ± 539.9	27.1 ± 4.9
*P*-value	0.411	0.176
A1A1	1122.0 ± 430.8	25.3 ± 4.3
A2A2	816.4 ± 366.7	27.1 ± 3.4
*P*-value	0.027^*^	0.176

Table 4 compares the ORs of *LPL*-Hind lll and *APO CII*-Ava ll polymorphism genotypes in all participants. The OR of H+H+ versus H−H− (10.5) was higher than that observed in H+H− versus H−H− (6.66). Regarding *APO Cll*-Ava ll, a lower OR was observed for A1A2 versus A2A2 (2.14) compared to A1A1 versus A2A2 (6.42).

**Table 4:** Comparison of LPL-Hind lll and APO CII-Ava ll polymorphism genotypes in all participants. Data are represented as odds ratio and 95% CI. ^*^P ≤ 0.05. 95% CI, 95 percentile confidence interval; LPL, lipoprotein lipase; APO Cll, apolipoprotein Cll

Genotype	Odds ratio	95% Cl	*P*-value
LPL			
H+H− vs. H−H−	6.66	1.21-36.74	0.014^*^
H+H+ vs. H−H−	10.50	2.010-54.83	0.002^*^
H+H+ and H+H− vs. H−H−	8.85	1.76-44.42	0.004^*^
APO Cll			
A1A2 vs. A2A2	2.14	0.78-5.82	0.067
A1A1 vs. A2A2	6.42	2.09-19.71	0.005^*^
A1A2 and A1A1 vs. A2A2	3.57	1.47-8.62	0.002^*^

Table 5 presents the relationships between TG and HDL-c levels and both* LPL*-Hind lll and *APO Cll*-Ava ll polymorphisms in all participants. For* LPL*-Hind lll, the mean serum HDL-c level was significantly lower in the H+H+ polymorphism group than in the H+H− group (*P*=0.002). For *APO Cll*-Ava ll, TG levels were significantly higher in the A1A1 polymorphism group compared to the A2A2 polymorphism group (*P *= 0.005).

**Table 5:** Triglycerides and HDL-c levels stratified by LPL-Hind lll and APO Cll-Ava ll polymorphisms in all participants. Data are represented as mean ± SD. ^*^P ≤ 0.05. SD, standard deviation; HDL, high-density lipoprotein; LPL, lipoprotein lipase; APO Cll, apolipoprotein Cll

Genotype	Triglycerides (Mean ± SD) (mg/dL)	*P*-value	HDL-cholesterol (Mean ± SD) (mg/dL)	*P*-value
*LPL*-Hind lll				
H+H−	536.4 ± 687.9	0.613	37.2 ± 13.8	0.323
H-H−	659.0 ± 574.9		32.7 ± 5.6	
H+H+	765.7 ± 515.0	0.553	30.3 ± 7.4	0.332
H−H−	659.0 ± 574.9		32.7 ± 5.6	
H+H+	765.7 ± 515.0	0.076	30.3 ± 7.4	0.002^*^
H+H−	536.4 ± 687.9		37.2 ± 13.8	
*APO Cll*-Ava ll				
A1A2	668.9 ± 614.3	0.182	33.4 ± 10.3	0.933
A2A2	487.1 ± 452.0		33.6 ± 8.7	
A1A1	890.2 ± 577.4	0.002^*^	30.9 ± 12.1	0.300
A2A2	487.1 ± 452.0		33.6 ± 8.7	
A1A2	668.9 ± 614.3	0.130	33.4 ± 10.3	0.365
A1A1	890.2 ± 577.4		30.9 ± 12.1	

## Discussion

To our knowledge, this was the first prospective genotypic investigation of the association between polymorphisms of the *LPL*-Hind III and *APO Cll*-Ava ll genes and hypertriglyceridemia in the Kurdish population in Duhok City, Iraq. Our results showed a significant difference in the genotypic and allelic frequencies of H+H+ between study groups, and a similar pattern was observed for A1A1. The results confirmed an association between H+H+ polymorphism and hypertriglyceridemia, as the OR of H+H+ vs. H−H− was approximately two times higher than that of H+H− vs. H−H− These results agreed with those of previous studies [12]. The H+H+ *LPL*-Hind lll polymorphism was the most frequent allele pair in patients with severe hypertriglyceridemia, as compared to those with normotriglyceridemia, indicating its role as a genetic indicator of vulnerability to severe hypertriglyceridemia in the Kurdish population.

Importantly, a strong association between H+H+ *LPL*-Hind lll polymorphism and TG levels has been observed in several populations [13]. Daoud et al. reported a significant link between TG levels and *LPL* gene polymorphism [14]. Williams observed high-serum TG concentrations among individuals with a high frequency of *LPL*-Hind lll polymorphism [15]. Lacey et al. supported this observation, reporting that TG level was strongly associated with *LPL* gene polymorphism even after adjustment for potential risk factors [16].

According to our statistical analysis, the H+H+ *LPL*-Hind lll polymorphism was the strongest genetic marker of susceptibility to severe hypertriglyceridemia, which was similar to the results of other studies [17]. The effect of the *LPL*-Hind III polymorphism on lipid parameters has not demonstrated the same consistency across other populations. In our study, we found an association between *LPL*-Hind III polymorphism and high lipid levels among the study population. This same association has been noticed in some populations but not others [4]. For example, Al-Samawi and Smaism reported an association between the H+H+ genotype of *LPL*-Hind III polymorphism and high TG levels in the Caucasian population of West Siberia [4], whereas others observed no significant differences in serum lipid levels in those with the H+H+, H+H−, and H−H− genotypes of *LPL*-Hind III polymorphism [13].

Regarding *APO Cll*-Ava ll, we observed a lower OR for A1A1 vs. A2A2 compared to A1A2 vs. A2A2, indicating that *APO Cll*-Ava ll gene polymorphism is a less significant genetic marker compared to H+H+ *LPL*-Hind III polymorphism, as reported elsewhere [4]. Recent evidence supports the association between *LPL*-Hind III polymorphism and both hemorrhagic stroke risk and high lipid levels in the Chinese Han population [18]. According to a study conducted in the western region of Saudi Arabia, coronary artery disease patients with Hind III genotypes had significantly higher TG, TC, and LDL-c values than controls among other genotypes. Furthermore, similar to our study, the Hind III genotypes were associated with markedly lower HDL-c levels. Both studies identified a link between Hind III polymorphism and elevated serum levels of TC, TG, and LDL-c, together with low levels of HDL-c [14]. Other evidence has also demonstrated that LPL genetic diversity plays a significant role in modulating serum HDL-c and TG levels, as well as the risk of ischemic heart disease [19].

Limitations

This study has certain limitations. The data obtained from subjects living in Duhok City may not represent all Kurds living in other parts of the Kurdistan region. Furthermore, identification of the *APO A5* (e.553G>T) and *APO B* genes was not conducted. Despite the lack of *APO A5* and *APO B* frequencies, this study demonstrated an association between *LPL*-Hind III polymorphism and hypertriglyceridemia, which renders Kurdish patients more susceptible to hypertriglyceridemia.

## Conclusions

Our results indicated that genotypes characterized by the H+H+ LPL-Hind III polymorphism may be hereditary indicators of vulnerability to hypertriglyceridemia in the Kurdish population. Furthermore, we found that the H+H+ and H+H− genotypes occurred more frequently in those with severe hypertriglyceridemia than in those with normotriglyceridemia. Therefore, a large genetic study across many populations, especially on H+H+ LPL-Hind lll polymorphism as a genetic marker of susceptibility to severe hypertriglyceridemia, may be of great importance.
